# Reduced Effectiveness of Contraceptive Implants for Women Taking the Antiretroviral Efavirenz (EFV): Still Good Enough and for How Long?

**DOI:** 10.9745/GHSP-D-15-00356

**Published:** 2015-12-15

**Authors:** James D Shelton

**Affiliations:** ^a^​ Global Health: Science and Practice, Editor-in-Chief, Washington, DC, USA.

## Abstract

EFV clearly reduces effectiveness of implants. However, the reduced effectiveness still appears better compared with short-acting methods overall, at least for the initial period of implant use, and may be acceptable to many women. We need better data on effectiveness, especially over the long term and on whether ENG implants (Implanon) might be more effective than LNG implants (Jadelle). Communicating the risk of pregnancy to clients under these circumstances is very challenging. In the longer term, providing an alternative to EFV, such as dolutegravir, might solve this problem.

## WHY REDUCED EFFECTIVENESS OF IMPLANTS IS AN IMPORTANT PROBLEM

The antiretroviral (ARV) efavirenz (EFV) is now recommended for first-line antiretroviral therapy (ART) by the World Health Organization (WHO).^1^ And since WHO now recommends ART for all people living with HIV,^2^ that makes virtually all the some 13 million women in sub-Saharan Africa living with HIV^3^ candidates for extended EFV use.

At the same time, contraceptive implants have many attractive features^4^ and are the fastest growing method of contraception in sub-Saharan Africa—taking a markedly increasing share of the contraceptive method mix. The reduced effectiveness of implants due to an interaction with EFV could result in many unwanted pregnancies among vulnerable women and undermine confidence in an outstanding contraceptive method.

## HOW DOES EFV DECREASE EFFECTIVENESS OF IMPLANTS?

**By reducing contraceptive hormone levels.** The very high contraceptive efficacy of implants comes from consistent release of low but highly effective levels of progestin in the blood. However, EFV speeds up the normal degradation of contraceptive progestins including those in implants (though not that of the injectable DMPA), lowering the progestin blood levels by roughly *half*.^5^^–^^9^ Because blood levels are already quite low, such a large reduction can lead to levels below the threshold at which the implant’s typically very high effectiveness is assured. Moreover, the progestin blood levels with implants are highest very soon after insertion and normally decline over the multiple years of an implant’s use. Thus, with continued EFV (and implant) use, these still lower progestin levels are **expected to increase the risk of pregnancy over time.**

The antiretroviral efavirenz speeds up the degradation of progestins found in contraceptive implants.

Risk of pregnancy with continued EFV and implant use is expected to increase over time.

## HOW MUCH DOES EFV REDUCE EFFECTIVENESS OF IMPLANTS?

Implants are normally extremely effective with a failure rate of less that 1% per year.^10^ Although the available studies on effectiveness of implants among women on EFV are limited, as shown in the Table, pregnancy rates for women on EFV are well above 1%. (Note data from the single Patel study^11^ are shown separately for the 2 types of implants.) The one exception is the small study of 25 women from Brazil,^14^ which found no pregnancies. Otherwise, the rates range from about 6% to 15%.

Pregnancy rates in women using EFV and implants generally range from 6% to 15%.

**TABLE.** Pregnancy Rates in Studies of Contraceptive Implants and the Antiretroviral Efavirenz

Implant Type and Study	Methodology	No. of Women	No. of Pregnancies	Pregnancy Rate (95% CI)	Period of Use
**LNG**					
Patel^11^	Retrospective electronic database	191^a^	6	7.1 (1.5, 12.6)	Unknown
Perry^12^	Retrospective chart review	121	15	10^a^	16.4 months
Scarsi^13^	Prospective clinical	20	3	15	48 weeks
**ENG**					
Patel^11^	Retrospective electronic database	641^a^	15	5.5 (2.5, 8.4)	Unknown
Kreitchmann^14^	Prospective clinical	25^b^	0	0	3 years
**Unknown**					
Pyra^15^	Secondary analysis of prospective study	9^a^	1	6	Unknown

Abbreviations: CI, confidence interval; EFV, efavirenz; ENG, etonogestrel; LNG, levonorgestrel.

^a^​ Estimated from data in publication.

^b^​ Believed to be predominantly EFV users.

## MIGHT ENG IMPLANTS BE MORE EFFECTIVE THAN LNG IMPLANTS?

The 2 leading implants are the single-rod Implanon, which releases the progestin etonogestrel (ENG), and the 2-rod Jadelle, which releases levonorgestrel (LNG). The primary mechanism for ENG and LNG implants is suppressing ovarian activity. Both are very highly effective, but the ENG implant is more effective than the LNG implant in suppressing ovarian activity.^16^^,^^17^

For women taking EFV, the results presented in the Table suggest better pregnancy prevention for the ENG implant than for the LNG implant, with failure rates from 0% to 6% versus 7% to 15%, respectively. On the other hand, in the large retrospective study by Patel^11^ based on electronic records of clinic visits, while the failure rate was a bit better for those using the ENG implant (5.5%) than the LNG implant (7.1%), the rates are fairly similar. However, even in that study, the numbers of pregnancies, particularly for the LNG implant, were very few and confidence intervals very large, so this study result is still compatible with a substantial difference in effectiveness.

For women taking EFV, the ENG implant might be more effective than the LNG implant.

## WHAT ABOUT THE EXPECTED INCREASE IN PREGNANCY RATES IN THE LATER YEARS OF IMPLANT USE WITH EFV?

Unfortunately we are largely in the dark, except that the pregnancy rates are bound to increase with longer duration of use. The blood level data suggest gradually declining levels of progestin over time, but that provides little insight. Only the small Brazil study^14^ has data for as many as 3 years. In the Patel study,^11^ information on duration was not available in the electronic database. But use of implants most probably tended to be early use, since implant use has only been rapidly scaling-up in recent years.

## FOR WOMEN TAKING EFV, HOW DOES THE REDUCED EFFECTIVENESS WITH IMPLANTS COMPARE WITH OTHER CONTRACEPTIVE METHODS?

For the initial time period at least, still generally better overall than the short-acting methods of oral contraceptives and injectables. The Patel study also assessed failure rates with other contraceptive methods for women taking EFV and found considerably higher failure rates with women using oral contraceptives and injectables compared with implants, though of course lower failure rates with IUDs and permanent methods.^11^ That higher risk with the short-acting methods was probably largely due to inconsistent use of pills and injectables. However, it is possible some of the women who reported use of a short-acting method, as recorded in the electronic database, may then have discontinued to become pregnant intentionally. But that seems unlikely to affect the overall finding that pregnancy rates for women taking EFV were better with implants than with injectables or pills.

The reduced contraceptive effectiveness when taking EFV and implants is still generally better overall than effectiveness of short-acting methods.

## WHAT DOES THIS EVIDENCE IMPLY FOR RECOMMENDATIONS ON USE OF IMPLANTS FOR WOMEN ON EFV?

Use of implants for women taking any ARV continues to fall under WHO Category 2, which is the “generally use” category.^18^ And the current evidence supports that position. As Patel recommends, “… all HIV-positive women should be offered all currently available contraceptive methods, and counseled about failure rates when used with efavirenz-based ART.”^11^

Use of implants for women taking any ARV continues to fall under WHO Category 2—“generally use.”

## CONVEYING THIS EVIDENCE ON EFFECTIVENESS TO CLIENTS IS VERY CHALLENGING

The body of knowledge on the advantages and disadvantages of contraceptive methods is exceedingly complex, and realistically only the most important information can be conveyed to clients. Effectiveness is clearly important, but it is already difficult to convey. And our limited and imprecise evidence on effectiveness of implants for women on EFV, especially over the long term, makes communicating it even more complex. Moreover, we don’t know if the ENG implant might be a better choice over the LNG implant. Personally, if I were such a client who wanted an implant, if given the choice, I would likely select an ENG implant, since the effectiveness is unlikely to be worse and might be better. But of course other factors weigh in on any individual’s choice. Clearly we need more evidence.

## NEW ARVS A POSSIBLE LONGER-TERM SOLUTION

One way out of this dilemma would be replacing EFV with another ARV that did not significantly reduce progestin blood levels. For example dolutegravir—an integrase inhibitor—has a number of advantages over EFV including apparently avoiding the way EFV reduces progestin blood levels.^19^^–^^21^ US guidelines already recommend such integrase inhibitors for first-line therapy, and EFV has been demoted to an alternative regimen.^22^ Dolutegravir is not widely available in developing countries as yet, though processes are in place hopefully to make it so in the coming years. Meanwhile, if and when it becomes available, preference for providing it to women choosing and using implants makes considerable sense.
